# Treatment of Trigeminal Zoster–Associated Pain With Burst and Tonic Trigeminal Ganglion Stimulation: A Retrospective Study

**DOI:** 10.1155/prm/3803644

**Published:** 2026-04-30

**Authors:** Hong Liu, Peng Huang, Liqun Huang, Xiaohong Jin, Fuhai Ji

**Affiliations:** ^1^ Department of Anesthesiology and Pain, The First Affiliated Hospital of Soochow University, Suzhou, China, sdfyy.cn

**Keywords:** burst, neuromodulation, trigeminal ganglion stimulation, trigeminal zoster–associated pain

## Abstract

**Purpose:**

Evidence regarding the efficacy of trigeminal ganglion stimulation for refractory trigeminal zoster–associated pain (TZAP) is limited. This study investigated the use of percutaneous trigeminal ganglion stimulation in a series of TZAP patients and evaluated the efficacy of burst trigeminal ganglion stimulation (bTGS) and tonic trigeminal ganglion stimulation (tTGS) modalities on pain relief.

**Patients and methods:**

We retrospectively reviewed all eligible TZAP patients who received temporary trigeminal ganglion stimulation in our department between January 2022 and August 2024. Spontaneous pain and mechanical allodynia intensity were assessed using the numeric rating scale (NRS) before treatment and at 1 week, the day of electrode removal, 1 month, 2 months, and 3 months post‐treatment. Other symptoms, pain interference with sleep, and scores on the self‐rating depression scale and self‐rating anxiety scale were also recorded.

**Results:**

Among 31 patients, 16 received tTGS and 15 received bTGS. Compared with baseline, there was a statistically significant decrease in spontaneous pain NRS scores at 3 months (mean difference, 5.0; 95% CI, 4.2–5.8; *p* < 0.001). Similarly, the proportion of patients with mechanical allodynia NRS scores 3 or less increased significantly at 3 months (difference in proportions, 61.3%; 95% CI, 42.2%–80.4%; *p* < 0.001). In the tTGS group, the mean spontaneous pain NRS score decreased from 8.1 (95% CI, 7.5–8.8) at baseline to 3.2 (95% CI, 1.8–4.6) at 3 months (*p* < 0.001). In the bTGS group, the mean spontaneous pain NRS score decreased from 7.3 (95% CI, 6.6–8.1) at baseline to 2.3 (95% CI, 1.2–3.3, *p* < 0.001) at 3 months (*p* < 0.001). No serious adverse events occurred.

**Conclusion:**

This retrospective study suggests that trigeminal ganglion stimulation is a promising therapeutic option for patients with TZAP and that bTGS may serve as a feasible alternative.

## 1. Introduction

When the varicella‐zoster virus lying dormant in the trigeminal ganglion (TG) is reactivated, it manifests as rash and pain along the trigeminal nerve branches, accounting for approximately 15%–20% of all herpes zoster cases [1]. Trigeminal zoster–associated pain (TZAP) often presents with moderate to severe intensity and responds poorly to conventional oral medications, nerve blocks, and neural radiofrequency treatments. Persistent pain profoundly impacts patients′ social functioning and quality of life, causing a significant socioeconomic burden. Therapeutic strategies aim to relieve the pain and symptoms secondary to herpes zoster [2]. The TG is the largest sensory ganglion in the cranial nerves and contains the cell bodies of the three major branches of the trigeminal nerve: the ophthalmic nerve (V1), the maxillary nerve (V2), and the mandibular nerve (V3). It plays an important role in peripheral and central sensitization associated with TZAP [3]. Supraorbital nerve stimulation has achieved efficacy in the treatment of zoster‐associated pain involving the ophthalmic nerve; however, pain coverage has been inadequate when lesions in other branches are present. Therefore, the TG has gradually become a therapeutic target for TZAP in recent years, but the literature is limited to case reports with traditional tonic stimulation [4, 5]. It seems to be a safe and effective treatment. The electrical impulses in traditional tonic stimulation are delivered in the form of regular biphasic square waves. This type of stimulation generates perceptible paresthesia corresponding to the innervation area of the target nerve, often described as a tingling or numbness sensation, which for some patients can be bothersome and may limit its application. Burst stimulation is a novel, paresthesia‐free stimulation pattern in which the impulses are delivered in bursts (short trains separated by a gap), mimicking the natural firing pattern of the neurons [6]. Burst spinal cord stimulation has been shown in available studies to be non‐inferior to traditional tonic spinal cord stimulation [7]. So far, there are no reports on burst trigeminal ganglion stimulation (bTGS) for the treatment of TZAP. In our study, we report the outcomes of 31 patients treated with trigeminal ganglion stimulation (TGS) at The First Affiliated Hospital of Soochow University, including cases where bTGS was used successfully in clinical practice. We also seek to compare burst stimulation to conventional tonic trigeminal ganglion stimulation (tTGS) in this study, although the sample size is small.

## 2. Materials and Methods

### 2.1. Patients

**FIGURE 1:**
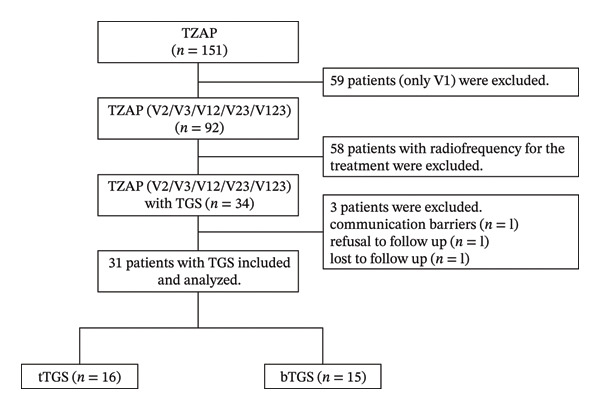
Flow diagram of the study patient selection. TZAP, trigeminal zoster–associated pain; V1, ophthalmic nerve; V2, maxillary nerve; V3, mandibular nerve; TGS, trigeminal ganglion stimulation; tTGS, tonic trigeminal ganglion stimulation; bTGS, burst trigeminal ganglion stimulation.

We included all adult TZAP patients (≥ 18 years) who underwent TGS procedures in our department between January 2022 and August 2024. All cases were classified as refractory due to achieving less than 50% pain relief from baseline, despite aggressive treatment with first‐line neuromodulatory agents for neuropathic pain (i.e., tricyclic antidepressants, pregabalin, and gabapentin) as well as invasive pain management strategies, including nerve blocks or pulsed radiofrequency of the peripheral trigeminal branches or TG. The concomitant use of second‐line agents (i.e., opioids, tramadol) was permitted in cases of intolerable adverse reactions (such as dizziness, nausea, and constipation). Patients who were unable to complete self‐evaluations, as well as those who were lost to follow‐up postoperatively, were excluded from the study (Figure 1). The study was approved by the Institutional Review Board of the First Affiliated Hospital of Soochow University.

### 2.2. Data Collection

We reviewed the medical records of patients with TZAP who underwent TGS in our department and collected the following data: patient background characteristics (age, sex, duration of pain, pain distribution, and previous therapy), procedure information (electrode placement site, stimulation parameters used, and electrode placement time), follow‐up information (spontaneous pain scores, mechanical allodynia scores, pain interference with sleep, other paresthesias, adverse events related to the treatment, self‐rating depression scale [SDS] and self‐rating anxiety scale [SAS] scores before treatment as well as after treatment at 1 week, the day of electrode removal). Follow‐up evaluations at 1 month, 2 months, and 3 months after treatment were carried out either by a telephonic or in‐person outpatient visit by an independent investigator who was not involved in the treatment. Mechanical allodynia was elicited by gently stroking the skin with a 2‐cm‐wide electric toothbrush (Philips HX6730), and the assessment procedure was standardized across all patients. Pain scores were documented using the numeric rating scale (NRS) score (range 0–10).

All 31 patients in our study completed follow‐up at all scheduled time points, with no data missing. The primary outcome for the study was the mean spontaneous pain NRS scores after treatment. Secondary outcomes included: (1) the proportion of patients exhibiting a minimum of 50% spontaneous pain relief on the NRS; (2) the proportion of patients with spontaneous pain scores of 3 or less; (3) the proportion of patients experiencing mechanical allodynia scores of 3 or less; (4) the proportion of patients reporting no sleep disturbance; and (5) mean SDS and SAS scores at all follow‐up time points.

### 2.3. Instruments

Puncture needle (Model: CTZ‐14, Shenzhen Qingyuan Medical Instrument Co., Ltd.). Percutaneous electrode kit for implantable neurostimulation system (Model: 3189, Abbott, USA): electrode diameter of 1.37 mm, containing 8 contacts, each contact length of 3 mm, and contact spacing of 4 mm.

### 2.4. Operative Procedure

All patients underwent implantation of the short‐term electrode under general anesthesia. Patients were placed in the supine position on the operating table, with a thin pillow positioned under their shoulders. Following disinfection and localization, the Hartel anterior approach was utilized for the puncture. The puncture needle was slowly and accurately advanced into the foramen ovale under lateral X‐ray guidance. The operator removed the stylet and inserted the electrode into Meckel’s cave. The depth of electrode placement was determined by each patient’s pain distribution. If the patient’s pain involved the first branch, contacts 0 and 1 of the electrode were placed over the slope, close to the trigeminal nerve root. Conversely, if only the second and third branches were affected, the electrode tip was not advanced past the slope. The puncture needle was then removed, and the electrode was secured to the patient’s cheek skin (Figure 2(a)). The electrode position was confirmed under X‐ray again postoperatively (Figures 2(b), 2(c), 2(d)). All electrode implantation procedures were conducted by a pain management physician with specialized training and over a decade of professional experience in interventional pain management.

FIGURE 2The fixation of electrode leads on the patient’s skin surface post‐operation (a) and intraoperative X‐ray illustrating the positioning of the electrode (b). lateral view; (c). anteroposterior view; (d). axial view).

**Figure:**
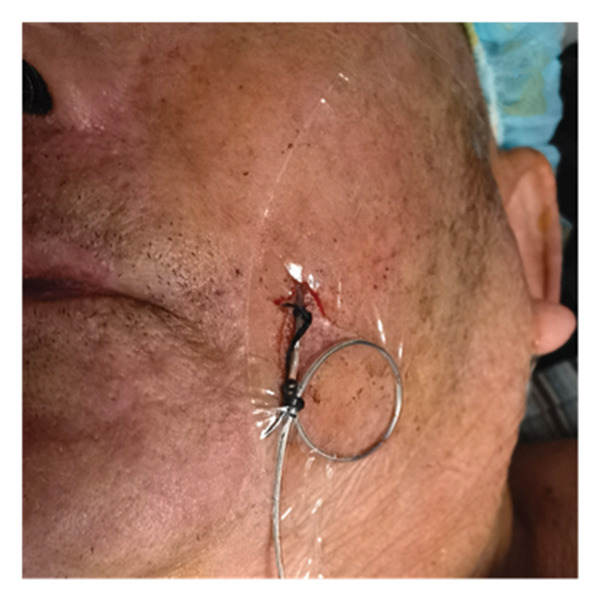
(a)

**Figure:**
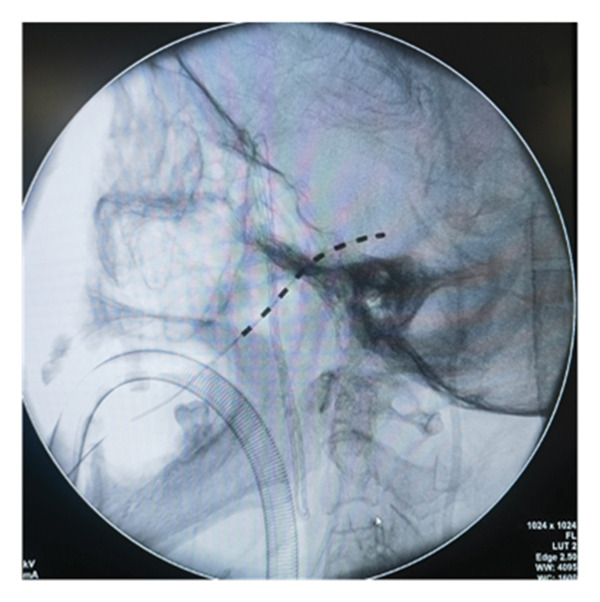
(b)

**Figure:**
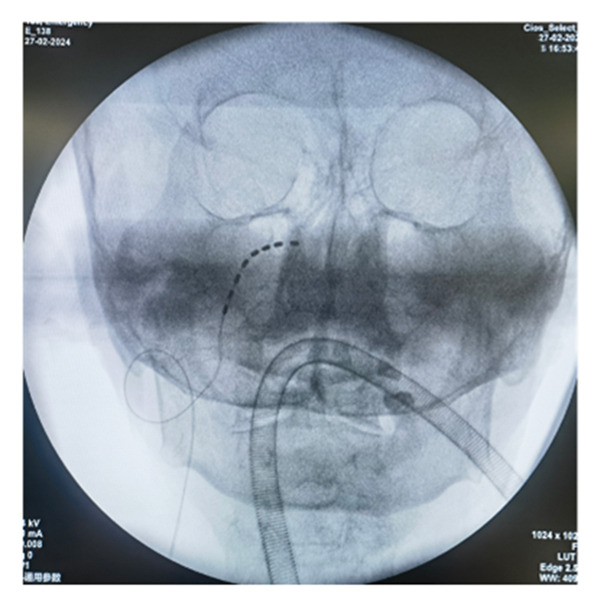
(c)

**Figure:**
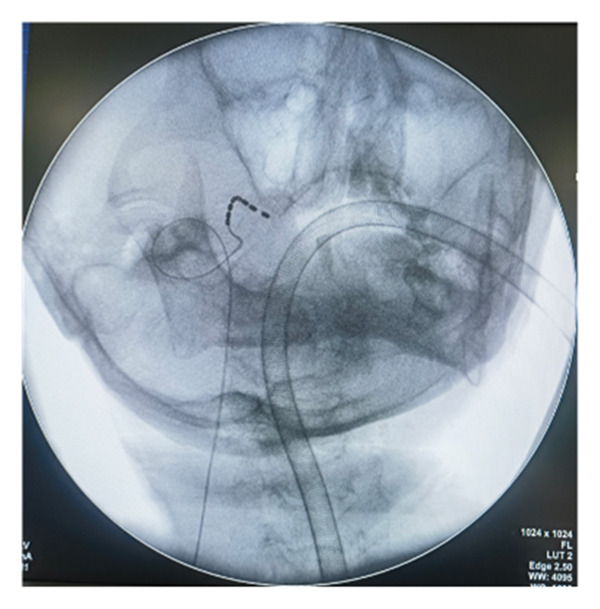
(d)

### 2.5. Programming

Following emergence from general anesthesia, contact polarity was initially adjusted using tonic mode, to ensure paresthesia covered more than 80% of the painful area. For patients assigned to tonic mode, maintenance stimulation was delivered using the following parameters: frequency 40–100 Hz, pulse width 200–400 µs, and amplitude titrated to patient comfort. Burst stimulation was approved for clinical use at our hospital in July 2023. All patients undergoing the procedure after this date received burst stimulation. For these patients, stimulation was switched to burst mode after following confirmation of contact polarity. The parameters were set as follows: burst frequency 40 Hz, intra‐burst frequency 500 Hz, pulse width 1000 µs, and amplitude ranging from 50% to 70% of the paresthesia perception threshold. Electrical stimulation was maintained for 21 days. Wound dressings were changed weekly to maintain a dry environment, and the electrode was removed after 21 days. Electrical stimulation was maintained for 21 days. Wound dressings were changed weekly to maintain a dry environment, and the electrode was removed on day 21.

### 2.6. Statistical Methods

Results are expressed as means (standard deviation, SD), medians (interquartile range, IQR), or frequencies (*n*, %), as appropriate for continuous and categorical variables. Patient characteristics were compared between the two cohorts using the two‐sample *t*‐test, Wilcoxon rank‐sum test, or Fisher’s exact test, as appropriate. Changes in pain scores are presented as means with 95% confidence intervals (CIs). To compare variables at different time points, a generalized estimating equation model was utilized. In all analyses, a two‐sided *p*‐value < 0.05 was considered statistically significant. Data analysis was performed using SPSS 27.0 (IBM Corporation, Armonk, NY, USA).

## 3. Results

### 3.1. Baseline Characteristics of Patients

Thirty‐one patients with TZAP were included in this study, with electrodes placed in the TG for 21 days. During the stimulation maintenance phase, 16 patients underwent tTGS and 15 patients received bTGS. The mean age of patients in the tTGS group was 71.9 (8.5) years, including 10 males (62.5%) and 6 females (37.5%). Among these patients, 1 patient had a disease duration of 1 month or less (6.3%), 14 patients had a disease duration between 1 and 3 months (87.5%), and 1 patient had a disease duration of 3 months or more (6.3%). The mean age of patients in the bTGS group was 70.9 (7.6) years, including 7 males (46.7%) and 8 females (53.3%). In the bTGS group, 2 patients had a disease duration of 1 month or less (13.3%), 10 patients had a disease duration between 1 and 3 months (66.7%), and 3 patients had a disease duration of 3 months or more (20.0%). Five patients in the tTGS group (31.3%) experienced pain involving the first branch of the trigeminal nerve, compared to 6 patients (40.0%) in the bTGS group. The remaining patients had pain localized to the second or third branches. Patient demographics are summarized in Table 1.

**TABLE 1:** Baseline characteristics for all patients.

Characteristic	tTGS (*n* = 16)	bTGS (*n* = 15)	*p* value
*Age, y*			
Mean (SD)	71.94 (8.45)	70.93 (7.59)	0.731

*Sex (n,%)*			
Male	10 (62.5)	7 (46.7)	0.479
Female	6 (37.5)	8 (53.3)	

*Duration of pain (n,%)*			
≤ 1 m	1 (6.3)	2 (13.3)	0.381
Between 1 m and 3 m	14 (87.4)	10 (66.7)	
≥ 3 m	1 (6.3)	3 (20.0)	

*Laterality (n,%)*			
Left	9 (56.3)	9 (60.0)	1.000
Right	7 (43.8)	6 (40.0)	

*Pain distribution (n,%)*			
V2	2 (12.5)	2 (13.3)	NA
V3	4 (25.0)	3 (20.0)	
V1V2	4 (25.0)	5 (33.3)	
V2V3	5 (31.2)	4 (26.7)	
V1V2V3	1 (6.25)	1 (6.7)	

*Note:* V1, ophthalmic nerve; V2, maxillary nerve; V3, mandibular nerve.

Abbreviations: bTGS = burst trigeminal ganglion stimulation, TGS = trigeminal ganglion stimulation, tTGS = tonic trigeminal ganglion stimulation.

### 3.2. Spontaneous Pain Scores

Significant reductions in spontaneous pain NRS scores were observed at all postoperative time points compared to baseline in both groups (*p* < 0.001). In the tTGS group, spontaneous pain NRS scores decreased from a baseline mean of 8.1 (95% CI: 7.5–8.8) to 3.2 (95% CI: 1.8–4.6, *p* < 0.001) at 3 months (Figure 3(a)). Specifically, 68.8% (11 of 16; 95% CI: 41.3%–89.0%, *p* < 0.001) achieved at least 50% spontaneous pain relief from baseline (Figure 3(b)) and 56.3% (9 of 16; 95% CI: 29.9%–80.2%, *p* < 0.001) reported spontaneous pain scores of 3 or less on the NRS (Figure 3(c)). In the bTGS group, spontaneous pain NRS scores decreased from a baseline mean of 7.3 (95% CI: 6.6–8.1) to 2.3 (95% CI: 1.2‐3.3, *p* < 0.001) at 3 months (Figure 3(a)). Specifically, 80.0% (12 of 15; 95% CI: 51.9%–95.7%, *p* < 0.001) achieved at least 50% spontaneous pain relief (Figure 3(b)) and 80.0% (12 of 15; 95% CI: 51.9%–95.7%, *p* < 0.001) reported spontaneous pain scores of 3 or less (Figure 3(c)). There was no significant difference in spontaneous pain relief between the tTGS and bTGS groups (*p* > 0.05). However, spontaneous pain NRS scores were numerically lower in the bTGS group compared to the tTGS group at the 3‐month follow‐up.

FIGURE 3Temporal pain alleviation quantified by the numeric rating scale (NRS). (a) Mean spontaneous pain NRS. (b) Proportion of patients with ≥ 50% spontaneous pain relief. (c) Proportion of patients with spontaneous pain NRS ≤ 3. (d) Proportion of patients with mechanical allodynia NRS ≤ 3. ^∗^ *p* < 0.05 vs. baseline, ^∗∗^ *p* < 0.001 vs. baseline, ^#^ *p* > 0.05 vs. tTGS, ^##^ *p* < 0.05 vs. tTGS.

**Figure:**
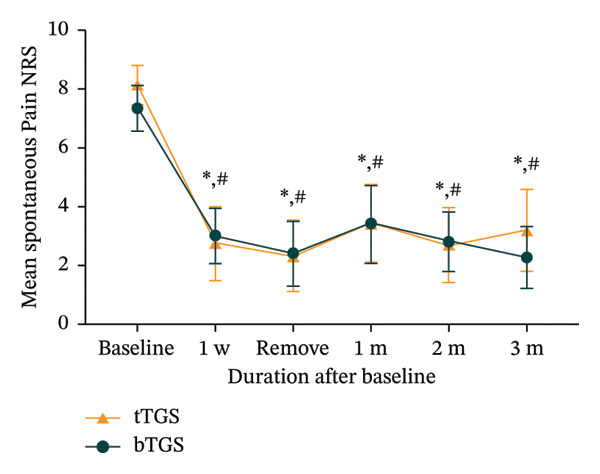
(a)

**Figure:**
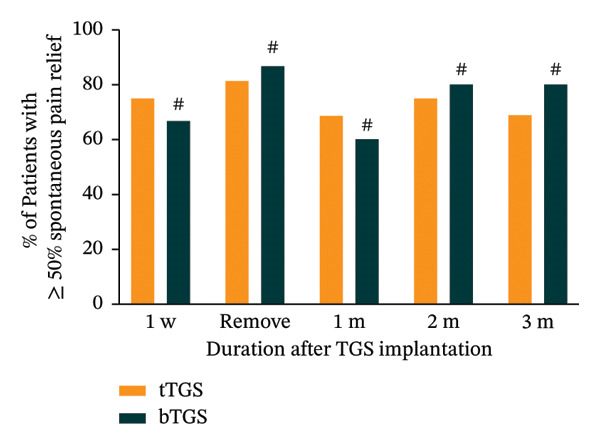
(b)

**Figure:**
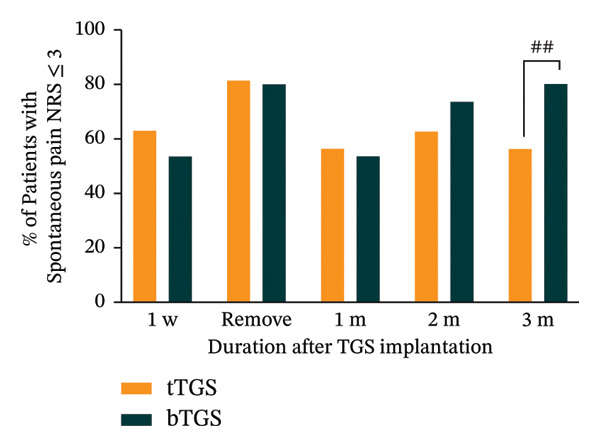
(c)

**Figure:**
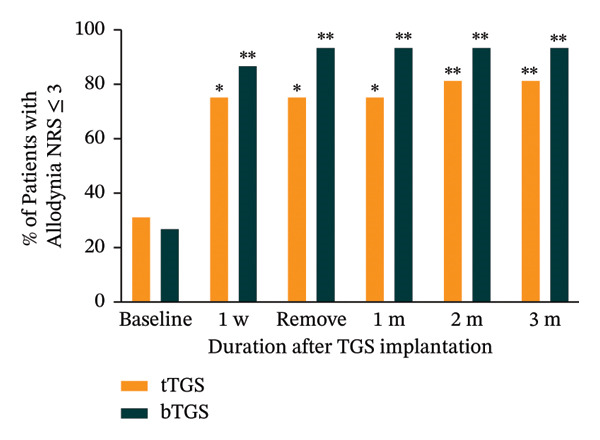
(d)

### 3.3. Mechanical Allodynia NRS Scores

During the postoperative period, both groups exhibited a significant reduction in mechanical allodynia, as measured by the NRS. The proportion of patients with mechanical allodynia NRS scores of 3 or less increased significantly in both groups (*p* < 0.05). At the 3 months follow‐up, the proportion of patients with mechanical allodynia NRS scores of 3 or less increased from a baseline of 31.3% (5 of 16; 95% CI: 11.0%–58.7%) to 87.5% (14 of 16; 95% CI: 61.7%–98.4%, *p* < 0.001) in the tTGS group. In the bTGS group, this proportion increased from 26.7% (4 of 15; 95% CI: 7.8%–55.1%) to 93.3% (14 of 15; 95% CI: 68.1%–99.8%, *p* < 0.001). Although the proportion of patients achieving relief was higher in the bTGS group across all postoperative follow‐up time points (Figure 3(d)), there was no statistically significant difference in mechanical allodynia relief between the tTGS and bTGS groups (*p* > 0.05).

### 3.4. Sleep Disturbance

**Figure 4:**
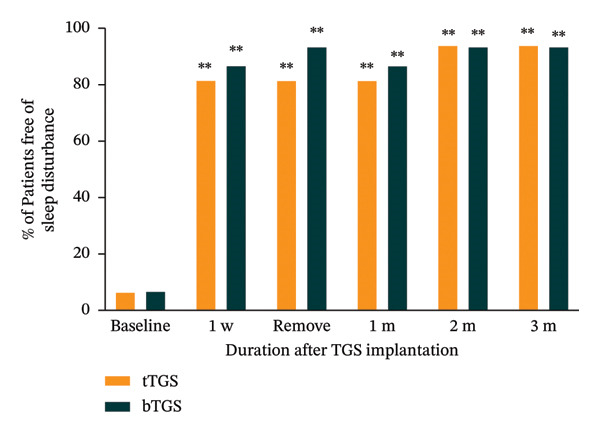
Proportion of patients free of sleep disturbance at baseline, 1 week, the day of electrode removal, 1 month, 2 months, and 3 months for the tTGS and bTGS groups. ^∗∗^ *p* < 0.001 vs. baseline.

Prior to treatment, only 6.3% (1 of 16; 95% CI: 0.2%–30.2%) of patients in the tTGS group and 6.7% (1 of 15; 95%CI: 0.2%–31.9%) in the bTGS group were free of sleep disturbances. However, this proportion increased to 81.3% (13 of 16; 95%CI: 45.6%–96.0%) in the tTGS group and 86.7% (13 of 15; 95% CI: 59.5%–98.3%) in the bTGS group at the 1‐week follow‐up, respectively. Importantly, significant improvements in sleep quality were observed at all postoperative time points, with a marked increase in the proportion of patients free of sleep disturbances compared to baseline (*p* < 0.001). There was no significant difference in the degree of improvement between the tTGS and bTGS groups (*p* > 0.05) (Figure 4).

### 3.5. SAS and SDS Scores

No significant differences were observed in SAS and SDS scores at 1‐week follow‐up when compared to baseline (*p* > 0.05). However, SAS and SDS scores decreased significantly at the day of electrode removal (*p* < 0.001). Furthermore, statistically significant improvements in SAS and SDS scores were found at other time frames when compared to the preoperative period (*p* < 0.05). At all time points, there was no significant difference in SAS and SDS scores between the two groups (*p* > 0.05) (Figure 5).

FIGURE 5Mean self‐rating anxiety scale (SAS) and self‐rating depression scale (SDS) scores assessing anxiety and depression severity in patients with trigeminal zoster–associated pain (TZAP). (a) SAS scores. (b) SDS scores. ^∗^ *p* < 0.05 vs. baseline, ^#^ *p* > 0.05 vs. baseline.

**Figure:**
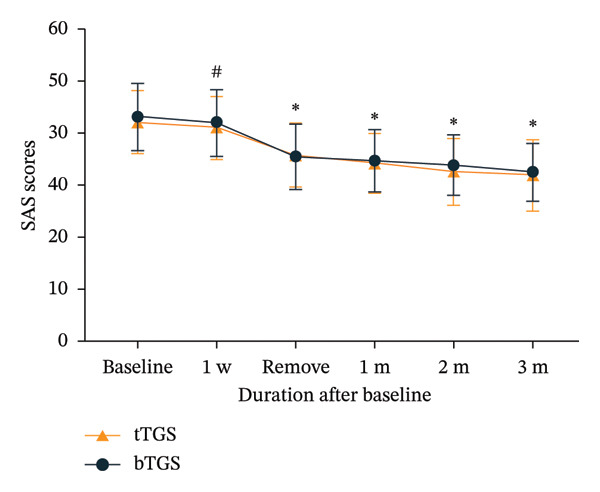
(a)

**Figure:**
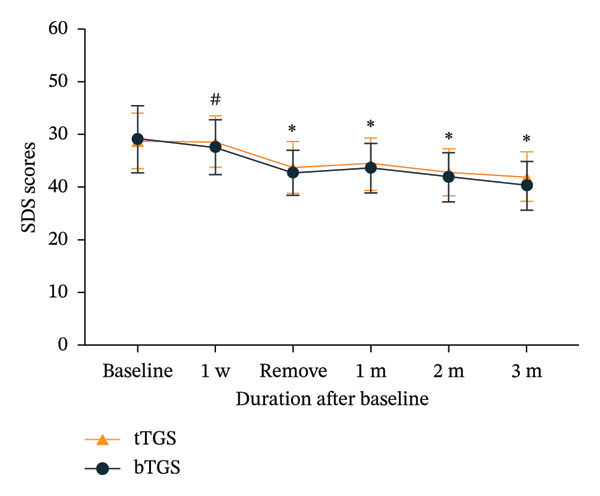
(b)

### 3.6. Other Paresthesias

In addition to pain, patients with TZAP may experience various paresthesias, such as itching, numbness, tightness, stiffness, and other forms of discomfort. These symptoms were observed in 20 patients (64.5%, 20 of 31) at baseline, and the proportion remained unchanged at the 3‐month follow‐up. In the tTGS group, the proportion of patients with moderate to severe paresthesia was 31.3% (5 of 16) at both baseline and the 3‐month follow‐up. Similarly, in the bTGS group, the proportion was 33.3% (5 of 15) at baseline and 26.7% (4 of 15) at the 3‐month follow‐up.

### 3.7. Analgesic Consumption

All patients had received aggressive first‐line medications (i.e., pregabalin, gabapentin, tricyclic antidepressants, and duloxetine) prior to TGS, with or without concomitant use of second‐line agents (i.e., opioids, tramadol). Subsequently, relevant analgesics were gradually tapered off starting 1 month postoperatively. The number of patients reducing medication reduction gradually increased in both groups at the 1‐, 2‐, and 3‐month follow‐ups. Specifically, in the tTGS group, opioid or tramadol usage decreased from a baseline of 62.5% (10 of 16; 95% CI: 35.4%–84.8%) to 31.3% (5 of 16; 95% CI: 11.0%–58.7%) at 3 months. Similarly, in the bTGS group, this proportion decreased from 66.7% (10 of 15; 95% CI: 38.4%–88.2%) to 33.3% (5 of 15; 95% CI: 11.8%–61.6%). At the 3‐month follow‐up, the proportions of patients who had completely discontinued analgesics were 25% (4 of 16; 95% CI: 7.3%–52.4%) in the tTGS group and 26.7% (4 of 15; 95% CI: 7.8%–55.1%) in the bTGS group. Nevertheless, a considerable proportion of patients still required pharmacological treatment, primarily consisting of anticonvulsants.

### 3.8. Adverse Events

Observed adverse events included pain induced by facial movement during electrode indwelling, cerebrospinal fluid leakage, and limited mouth opening. No patients experienced severe adverse events such as intracranial infection or hematoma. Electrode displacement was not observed. Specifically, facial pain and discomfort induced by facial movement during electrode indwelling occurred in 3 out of 16 patients (18.8%) in the tTGS group, and 3 out of 15 patients (20.0%) in the bTGS group. Notably, this pain was distinct from TZAP and was significantly relieved following electrode removal. Cerebrospinal fluid leakage occurred in 3 out of 16 patients (18.8%) in the tTGS group, and 2 out of 15 patients (13.3%) in the bTGS group during electrode removal. However, none of these patients continued to leak after compression bandaging at the puncture site. One patient (6.3%) in the tTGS group and one patient (6.7%) in the bTGS group developed symptoms of limited mouth opening after electrode placement, which persisted at the 3‐month postoperative follow‐up. The trismus was mild to moderate in severity and affected the patients′ quality of life.

## 4. Discussion

Therapeutic challenges persist in the clinical management of refractory zoster–associated pain. Although temporary spinal cord stimulation has significantly improved outcomes for spinal nerve‐associated zoster‐associated pain and has gradually become an important minimally invasive neuromodulation technique, its application is limited in cranial regions [8–10]. The trigeminal nerve is the cranial nerve most commonly affected by herpes zoster (57.9%) [11]. Despite its distinct anatomical location compared to spinal nerves, the pathophysiological process and pain mechanisms following varicella‐zoster virus invasion are similar [12, 13]. High cervical spinal cord stimulation has proven effective for trigeminal postherpetic neuralgia in several studies; however, this approach carries an inherent risk of lead displacement due to cervical motion [12–15]. Peripheral nerve stimulation, targeting the supraorbital and infraorbital nerves, has been utilized primarily for zoster‐associated pain in the V1 or V2 distributions, with limited application in the V3 region. However, infraorbital nerve stimulation often fails to provide complete paresthesia coverage of the entire V2 distribution [16, 17]. As a neuromodulatory technique, TGS has demonstrated efficacy in relieving TZAP. Xu et al. retrospectively analyzed six patients undergoing short‐term TGS, reporting effective pain relief in the V2 and V3 distributions [5]. Gupta documented a case of trigeminal postherpetic neuralgia where the patient maintained 50% pain relief following TGS [18]. Additionally, Niu et al. reported successful management of subacute TZAP using temporary TGS, with the visual analog scale decreasing from 9 preoperatively to 1 at electrode removal (14 days postoperatively) and increasing to 4 at 12‐month follow‐up [4]. In the present study, spontaneous pain NRS scores decreased significantly at the 3‐month follow‐up (mean difference, 5.0; 95% CI, 4.2–5.8; *p* < 0.001), consistent with these previous reports. Mechanical allodynia is another debilitating symptom in TZAP that severely impacts activities of daily living, such as face washing. Notably, the alleviation of mechanical allodynia via TGS has not been previously documented. This study provides evidence supporting the application of TGS in patients with significant mechanical allodynia.

Prior studies have not explored the use of bTGS for treating TZAP. To our knowledge, this study represents the first attempt to preliminarily compare the therapeutic effects of bTGS and tTGS in managing TZAP. Among patients receiving bTGS or tTGS, no substantial difference was observed in the relief of spontaneous pain or mechanical allodynia at the 3‐month follow‐up. Our finding that paraesthesia‐free bTGS can effectively reduce trigeminal zoster–associated spontaneous pain and mechanical allodynia intensity strongly suggests that paraesthesia associated with tonic stimulation may be not necessary for optimal temporary TGS outcomes. These findings are in accordance with early reports of good achieved with burst spinal cord stimulation for chronic pain [19–21].

After electrode removal, spontaneous pain NRS scores increased slightly in both stimulation modes at the 1‐month follow‐up, primarily due to pain rebound in some patients. In both the tTGS and bTGS groups, three patients each experienced a shift from painless or mild pain to moderate‐to‐severe pain. This observation is consistent with the increase in pain scores following SCS lead removal reported in herpetic neuralgia studies [8–10]. Notably, burst stimulation did not mitigate this phenomenon.

In addition to severe pain, herpes zoster also brings pruritus, numbness, tightness, stiffness, and other paresthesias, affecting the quality of life of patients. Regrettably, TGS does not seem to be effective in relieving these paresthesias caused by herpes zoster. In this study, no significant difference was observed in paresthesia severity before and after treatment, either in the tTGS group or in the bTGS group. Our study did not apply standard clinical assessments of neurological function, so paresthesias were classified as mild, moderate, or severe based solely on the patients’ self‐assessment. As such, assessor variability could be significant, and the neurological improvement findings should be interpreted in the context of this limitation. Further studies including more objective measures will be required to validate these observations.

Pain and paresthesias caused by herpes zoster can also negatively affect sleep quality. These symptoms are often worse at night, particularly for patients with TZAP. Sleep deprivation further exacerbates pain scores, affects mental health, and impairs daytime functioning. This study indicates that TGS significantly improves sleep quality in patients with TZAP. Additionally, patients reported improvements in emotional wellbeing, suggesting a broad positive impact of this treatment on their lives.

Although the Hartel anterior approach for electrode placement is well established, there is currently no consensus regarding the optimal depth of TGS electrode placement. Texakalidis et al. reported the distance from the foramen ovale to the petrous apex as 24.75 (3.34) mm [22]. In this study, we utilized SCS electrodes (Model 3189, Abbott, Inc.) with a contact length of 3 mm and an intercontact spacing of 4 mm. The first two contacts span a range of 1 cm. Our experience suggests that when a patient’s pain distribution involves the first branch, the electrode should be positioned over the slope with approximately two contacts, while maintaining a distance of approximately three or four contacts from the foramen ovale to the petrous edge (as confirmed by lateral radiograph). In this configuration, the first two contacts are close to the trigeminal nerve root, enabling electrical stimulation to effectively cover the first branch. Conversely, if the patient’s pain is limited to the second and/or third branches, the electrode tip should not need to extend beyond the slope. This positioning minimizes the risk of nerve injury and cerebrospinal fluid leakage.

In previous TGS studies, uncomplicated local infection and electrode displacement were the primary causes of treatment failure. Although the reported incidence of postoperative infection ranges from 18% to 37%, all patients in those studies received stage II permanent implantation and were cured following hardware removal and antibiotic therapy [22–24]. In the study by Taub et al., three patients experienced exacerbated sensory loss in the face, presumably due to injury to the trigeminal root, ganglion, or branches during stage I or II procedures [24]. Additionally, two patients developed transient diplopia, likely secondary to injury of the fourth or sixth cranial nerve during transcutaneous insertion. In this study, two patients developed the limited mouth opening after treatment. This symptom was unrelated to TGS parameter adjustments and showed no significant improvement at the 3‐month follow‐up. We hypothesize that this complication may be related to damage to the trigeminal nerve or its branches caused by the insertion procedure. Operators should handle tissues gently during the procedure, avoiding repeated punctures and minimizing electrode repositioning to reduce the risk of nerve injury.

Some patients showed pain and discomfort distinct from TZAP during facial movement, which may be attributed to nerve stimulation by the electrode. Symptoms were significantly relieved upon electrode removal, further supporting this hypothesis. Notably, this study utilized spinal cord stimulation electrode. The use of softer and slimmer, TG‐specific electrodes may enhance patient tolerance and comfort. Cerebrospinal fluid leakage following lead removal was another relatively common complication, likely resulting from dural puncture during the procedure, allowing CSF to escape along the puncture tract. Dural puncture increases the risk of infection. Therefore, attention to electrode placement depth is crucial. If the pain distribution involves the V1 branch, the electrode tip can cross the slope by one to two contact lengths. Conversely, for patients without V1 involvement, the electrode tip should not exceed the slope to minimize the risk of dural puncture.

## 5. Limitations

First, the trigeminal division predominantly affected by TZAP (over 75%) is the ophthalmic (V1), in contrast to the second or third divisions (V2, V3). This distribution posed significant challenges for sample collection in this study. Although we conducted an exploratory analysis of the effectiveness and safety of two different stimulation modes based on this small sample size, the limited sample size and retrospective design restrict our ability to draw causal inferences and potentially introduce bias. Second, this study included two non‐concurrent cohorts, as bTGS was adopted for all patients in the hospital following its approval in July 2023. However, this temporal distinction introduced potential selection bias and confounding factors, contributing to the limited statistical power observed when comparing the two groups. Third, the follow‐up period in this study was limited to 3 months post‐treatment for both stimulation modalities, lacking longer‐term outcomes. Furthermore, we did not employ any direct patient‐reported outcome measures (PROMs) related to device tolerability and subjective comfort during stimulation. Consequently, we are unable to adequately interpret the differences in patient experience between the two modes. Finally, no quality‐of‐life scores or Pittsburgh sleep quality index (PSQI) assessments were recorded prior to or following TGS in our cohort. Therefore, a prospective, randomized, controlled trial with a larger sample size is warranted to compare the relative efficacies and safety of these two stimulation modes [25].

## 6. Conclusion

Our study demonstrates the utility of TGS as a viable treatment option for patients with TZAP, thereby contributing to the existing body of evidence. TGS achieves a significant reduction in pain intensity. Additionally, this study suggests a promising clinical therapeutic effect of bTGS. Further studies are warranted to confirm these findings and to establish the non‐inferiority of burst stimulation compared to conventional tonic stimulation.

## Author Contributions

**Hong Liu**: data curation (equal); formal analysis (equal); investigation (equal); project administration (equal); writing–original draft (lead). **Peng Huang**: data curation (equal); formal analysis (equal); project administration (equal); writing–original draft (equal). **Liqun Huang**: data curation (equal). **Xiaohong Jin**: data curation (equal); formal analysis (equal); investigation (equal); project administration (equal); writing–review and editing (equal). **Fuhai Ji**: data curation (equal); formal analysis (equal); investigation (equal); project administration (equal); writing–review and editing (equal).

## Funding

This work was supported by the National Natural Science Foundation of China (82471281), Key Medical Research Projects in Jiangsu Province (ZD2022021), Key R&D Program Projects in Jiangsu Province (BE2023709), Suzhou Key Laboratory of Anesthesiology (SZS2023013), Suzhou Clinical Medical Center for Anesthesiology (Szlcyxzxj202102), and National Clinical Key Specialty for Anesthesiology.

## Ethics Statement

Ethical approval was granted by the Ethics Committee of the First Affiliated Hospital of Soochow University (Approval Number: [2024] Ethics Approval No. 561). Written informed consent was obtained from all participants prior to data collection, including consent for the publication of the image in Figure 2.

## Conflicts of Interest

The authors declare no conflicts of interest.

## Data Availability

All raw data and code are available upon request.
